# Lost in Translation: Matching on Love Languages Explains Little Variation in Relational Outcomes in Mid and Late Life

**DOI:** 10.1093/geroni/igaf122.1395

**Published:** 2025-12-31

**Authors:** William Chopik, Louis Hickman, Rebekka Weidmann, Mariah Purol, Hyewon Yang

**Affiliations:** Michigan State University, East Lansing, Michigan, United States; Virginia Tech, Blacksburg, Virginia, United States; Brigham Young University, Provo, Utah, United States; Union College, Schenectady, New York, United States; Michigan State University, East Lansing, Michigan, United States

## Abstract

Love languages—and the degree to which people “match” on them—have captivated lay audiences searching for explanations for why some relationships do or do not work out. Previous work formally testing whether matching on love languages translates to better relationship success has been relatively rare, provided mixed results, and has tested matching in non-optimal ways. In the current study, we examined whether methodological variation in matching procedures (e.g., difference scores, response surface analysis, and machine learning) can help characterize whether love languages have implications for relationship success in 954 male/female couples (Mage = 55.42, SD = 16.07; MRelationshipLength = 27.39 years). Across all these approaches and all possible love language pairings (via machine learning methods), we found virtually no evidence that matching on love languages explained additional variation in outcomes above and beyond individual and spousal personality traits and attachment orientations. Further, expression (highest r = .795) and preferences (highest r = .774) between love languages were occasionally and problematically, highly correlated. Based on the results of the current study, it is worth revisiting whether love languages tap into distinct relationship preferences and behaviors or if they reflect broader responsiveness or positive relationship behaviors regardless of matching. The presentation will conclude with a critical discussion of the personal and relational characteristics most likely to lead to resilience in the context of close relationships.

